# Supplementary material for: Insomnia, Cognitive Impairment, or a Combination of Both, Alter Lipid Metabolism Due to Changes in Acylcarnitine Concentration in Older Persons
Source: Metabolites. 2025 Jun 19;15(6):417. doi: 10.3390/metabo15060417 (PMC12195264; doi:10.3390/metabo15060417)
Supplement: Supplementary file 1 [file metabolites-15-00417-s001.zip › metabolites-3687710-supplementary.pdf]

Supplementary Information

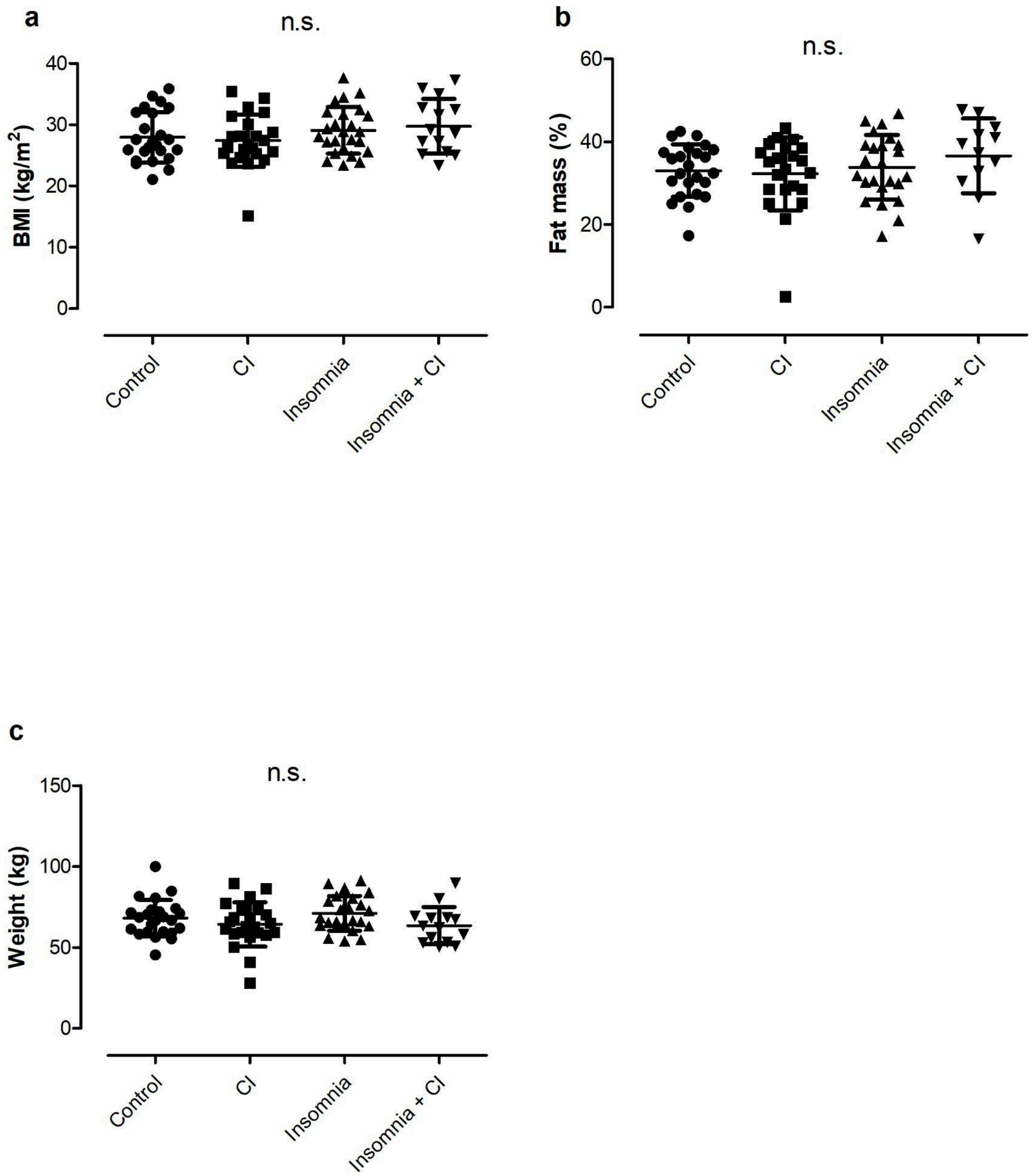

Figure S1. Comparison of variables that could potentially impact our metabolomic analysis. These plots do not show any statistically significant differences between groups in the main variables that may impact on the metabolomic analyses. (**a**) Plot corresponding to BMI (kg/m2), (**b**) Plot corresponding to Fat mass (%), and (**c**) Plot corresponding to Weigh (kg)t. Data were analyzed using One-way ANOVA followed by a Dunn's post hoc test. p-Value<0.05, non-significant (n.s.).

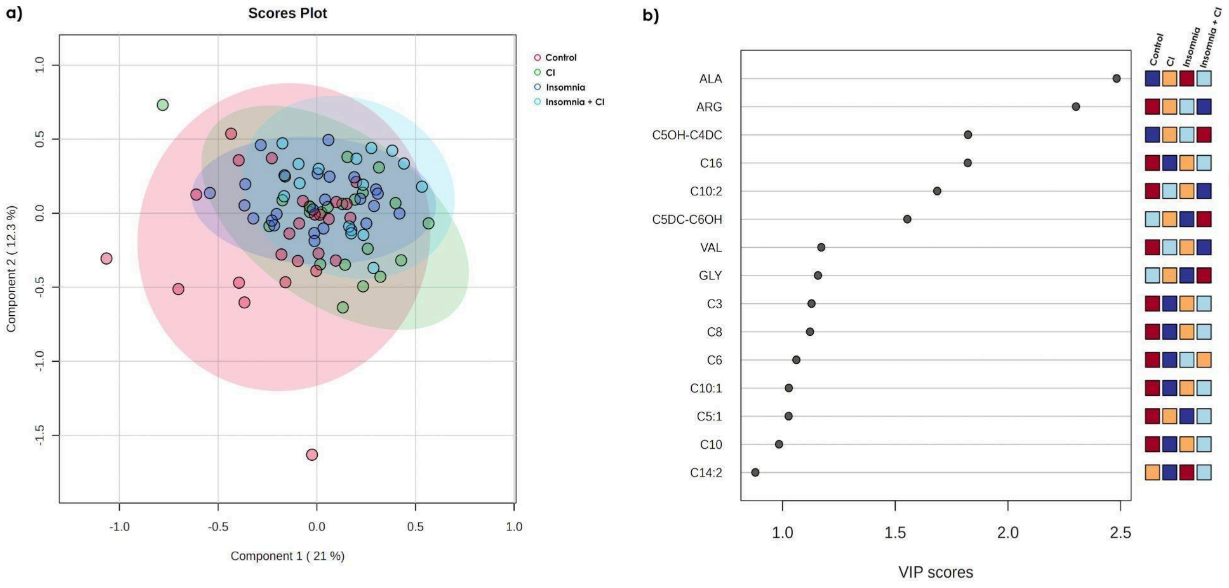

Figure S2. Comparison of metabolic profiles in serum samples derived from older persons with Insomnia, CI, Insomnia + CI and Controls. (a) PLS-DA analysis of relative concentration occurring in serum metabolome in the control group (red), CI (green), Insomnia (purple) and Insomnia + CI (blue). The proportion of variance corresponds to Component 1: 21% and Component 2: 12.3%, Accuracy: 0.28301, R2: 0.10064, Q2: -0.16206, and the permutation p-Val= 0.6205. **(b)** VIP analysis represents the relative contribution of metabolites to the variance between groups. The colour scale (on the right side) represents metabolite concentration ranging from low (blue) to high (red).The VIP score cut-off has been adjusted to 1.0.
